# Spatiotemporal Variability of Remotely Sensed PM_2.5_ Concentrations in China from 1998 to 2014 Based on a Bayesian Hierarchy Model

**DOI:** 10.3390/ijerph13080772

**Published:** 2016-08-01

**Authors:** Junming Li, Meijun Jin, Zheng Xu

**Affiliations:** 1 Institute of Geodesy and Geophysics, Chinese Academy of Sciences, Xudong Street 340, Wuhan 430077, China; lijunming_dr@l26.com; 2 College of Architecture and Civil Engineering, Taiyuan University of Technology, Yingze Street 79, Taiyuan 030024, China; 3 University of Chinese Academy of Sciences, No.19A Yuquan Road, Beijing 100049, China; 4 Henan University, Jin Ming Avenue, Kaifeng 475001, China; XuZheng_Geo@163.com

**Keywords:** PM_2.5_ concentrations, spatiotemporal variation, Bayesian hierarchy model

## Abstract

With the rapid industrial development and urbanization in China over the past three decades, PM_2.5_ pollution has become a severe environmental problem that threatens public health. Due to its unbalanced development and intrinsic topography features, the distribution of PM_2.5_ concentrations over China is spatially heterogeneous. In this study, we explore the spatiotemporal variations of PM_2.5_ pollution in China and four great urban areas from 1998 to 2014. A space-time Bayesian hierarchy model is employed to analyse PM_2.5_ pollution. The results show that a stable “3-Clusters” spatial PM_2.5_ pollution pattern has formed. The mean and 90% quantile of the PM_2.5_ concentrations in China have increased significantly, with annual increases of 0.279 μg/m^3^ (95% CI: 0.083−0.475) and 0.735 μg/m^3^ (95% CI: 0.261−1.210), respectively. The area with a PM_2.5_ pollution level of more than 70 μg/m^3^ has increased significantly, with an annual increase of 0.26 percentage points. Two regions in particular, the North China Plain and Sichuan Basin, are experiencing the largest amounts of PM_2.5_ pollution. The polluted areas, with a high local magnitude of more than 1.0 relative to the overall PM_2.5_ concentration, affect an area with a human population of 949 million, which corresponded to 69.3% of the total population in 2010. North and south differentiation occurs in the urban areas of the Jingjinji and Yangtze Delta, and circular and radial gradient differentiation occur in the urban areas of the Cheng-Yu and Pearl Deltas. The spatial heterogeneity of the urban Jingjinji group is the strongest. Eighteen cities located in the Yangtze Delta urban group, including Shanghai and Nanjing, have experienced high PM_2.5_ concentrations and faster local trends of increasing PM_2.5_. The percentage of exposure to PM_2.5_ concentrations greater than 70 μg/m^3^ and 100 μg/m^3^ is increasing significantly.

## 1. Introduction

Air pollution has become an important factor that threatens human health. Particulate matter (PM) suspended in the air is regarded as the most severe type of pollutant [1]. PM with an aerodynamic diameter of less than 2.5 μm (PM_2.5_) can enter deeply into the human body through breathing [2]. Some public health studies have found that PM_2.5_ affects public health significantly and adversely [3,4,5,6,7,8,9,10,11]. Chen et al. [12] reported that a 3-year reduction in average life expectancy and a 14% increase in total mortality would result from a respirable PM concentration increase of 100 μg/m^3^. Forouzanfar et al. [13] reported that PM_2.5_ resulted in three million premature deaths worldwide in 2013. Because PM_2.5_ can easily enter the human body, thereby causing chronic disease and increasing mortality and morbidity, PM_2.5_ has become an important issue in international pollution research [2]. In recent years, estimating ground-level PM_2.5_ concentrations by using satellite-based remote sensing data has become more important for studying long-term changes of PM_2.5_ concentrations due to advances in new retrieval algorithms that have led to high accuracy and long-term data stability [14,15,16,17,18,19,20,21]. PM_2.5_ concentrations estimated from satellite data can fill the gaps in data obtained from fixed sites and result in high-resolution data [22]. Compared with ground-based site monitoring, remote sensing is capable of quickly obtaining large-scale and spatially continuous PM_2.5_ concentrations [2,23]. Currently, not enough monitoring stations are available to fully capture the large scale variability of PM_2.5_ in China [11]. PM_2.5_ concentrations retrieved by satellites have been used as an effective method for studying the regional distribution and variation of air pollution [24].

With its industrial development and rapid urbanization, China is facing a challenging environmental pollution problem, particularly haze pollution [25]. Severe and widespread PM_2.5_ pollution in China has raised more and more public concern [26]. Based on the World Health Organization (WHO) recommendations, Chinese air does not currently meet air quality standards [27,28]. In China, ambient PM pollution was the fourth leading risk factor for death in 2010 [29]. Given the unbalanced development and diversity in China, the PM_2.5_ pollution in China likely exhibits strong spatial heterogeneity. If the spatiotemporal patterns of PM_2.5_ concentrations in China could be accurately determined, the resulting knowledge could be used to prevent and control PM_2.5_ pollution. In this paper, the spatiotemporal variations of PM_2.5_ in China from 1998 to 2014 are explored. Few researchers have studied the spatiotemporal patterns of PM_2.5_ concentrations in China. Lin et al. [10] studied the spatiotemporal variations of PM_2.5_ concentrations in China from 2001 to 2010 by using the global annual average PM_2.5_ grids data from the Battelle Memorial Institute and the Centre for International Earth Science Information Network/Columbia University. Ma et al. [28] estimated the PM_2.5_ concentrations from 2004 to 2013 in China from MODIS remote sensing data and briefly discussed the spatiotemporal trends of the PM_2.5_ concentrations. Peng et al. [2] systematically investigated the space-time patterns of PM_2.5_ concentrations in China from 1999 to 2011 using a resolution of 10 km and PM_2.5_ data retrieved by van Donkelaar et al. in 2015 based on classic statistical methods [30].

To our knowledge, we are the first to employ a space-time Bayesian hierarchy model to analyse the spatiotemporal variations of PM_2.5_ concentrations. The remotely sensed PM_2.5_ data used in the paper were obtained from the latest versions of the datasets from 1998 to 2014, which were estimated by van Donkelaar et al. [31]. In addition to the national scale, four leading urban areas in China have been studied. The objective of this study is to systematically explore spatiotemporal variations and trends in PM_2.5_ concentrations that can be used by Chinese policymakers or others to prevent or reduce PM_2.5_ pollution.

## 2. Data and Methods

### 2.1. Study Area and Data

**Figure 1:**
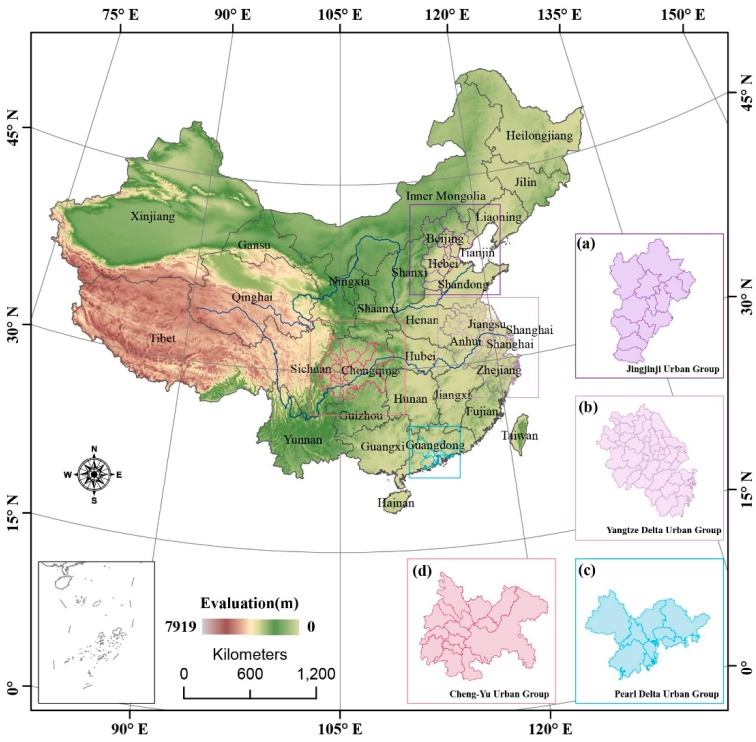
Chinese provincial administrative map with elevation and four large urban group areas: (**a**) Jingjinji urban group; (**b**) Yangtze Delta urban group; (**c**) Pearl Delta urban group; and (**d**) Cheng-Yu urban group.

China (104°21′15″ E, 28°42′30″ N) is located in east Asia, with high and low lying areas from east to west and a ladder-like distribution, as shown in Figure 1. The four basins and three plains account for approximately 33% of China’s surface [32]. In addition, this paper focuses on four large urban groups, which are shown in Figure 1, and include the Jingjinji urban group containing Beijing and Tianjin; the Yangtze Delta urban group containing Shanghai, Nanjing and Hangzhou; the Pearl Delta urban group containing Guangzhou, Hong Kong and Shenzhen; and the Cheng-Yu urban group containing Chengdu and Chongqing. Although these four urban groups cover of 9.26% of the total area of China, they account for 35.42% of the total population [33,34].

The remotely sensed PM_2.5_ concentration data product with a resolution of 0.1° × 0.1° used in this paper was downloaded from the Atmospheric Composition Analysis Group [35]. The dataset was produced by van Donkelaar et al. [31] from the Atmospheric Physics Institute of Dalhousie University in Canada. The remotely sensed PM_2.5_ concentration dataset was estimated by combining Aerosol Optical Depth (AOD) data retrieved from NASA MODIS, MISR, and SeaWIFS instruments with the GEOS-Chem chemical transport model. Subsequently, the dataset was calibrated to global ground-based observations of PM_2.5_ by using the Geographically Weighted Regression (GWR) method. The raw grids data with a resolution of 0.1° × 0.1° based on the WGS-84 projection were translated to the Asia Lambert Conformal Conic projection. Due to the effects of perennial clouds, snow-covered mountains, etc., data were missing from the remotely sensed PM_2.5_ concentration grids data. We used a focal statistic technique to spatially estimate the missing values by using a sliding average of 6 × 6 windows and repeating the process until no values were missing.

Besides the PM_2.5_ concentration grid data that were inferred from the remote sensing data, three other types of data, GIS vector data, elevation data with a resolution of 4 km × 4 km, and population data at the country and district levels, were used in this paper. The GIS vector data and elevation data were used to generate a base map, and the GIS vector polygons consisted of counties or districts as statistical geographical units. The GIS vector data are publically available from the National Geomatics Center of China. The elevation data were obtained from the ASTER GDEM [36] dataset. The two geographical datasets mentioned above were also translated to the Asia Lambert Conformal Conic projection. Two types of population data were used in this paper: population census data from the 6th National Population Census in the county or district administrative region and national population grid data with a resolution of 1 km × 1 km from 2000 and 2010 and provided by the Data Center for Resources and Environmental Sciences, Chinese Academy of Sciences (RESDC) [37]. Population data are mainly used for evaluating the proportions of the population exposed to high PM_2.5_ pollution.

### 2.2. Methods

#### 2.2.1. General Statistics

The overall temporal variations of PM_2.5_ concentrations in China and the four urban group areas during 1998–2014 were analysed mainly using traditional statistical methods. Linear regressions of the mean and 90% quantile of PM_2.5_ concentrations in China from 1998 to 2014 were used to assess the overall variation tendency of PM_2.5_ concentration at the national scale. Box plots of the PM_2.5_ concentrations in the four urban group areas were used to evaluate the temporal trends of PM_2.5_ pollution in the four large urban areas. In addition, a temporal histogram composed of the yearly histogram of PM_2.5_ concentrations in China was used to describe the temporal variability of the PM_2.5_ concentration distribution in China over 17 years.

#### 2.2.2. Space-Time Bayesian Hierarchal Model

Bayesian statistical inference integrates three types of information, population properties, sample information, and prior knowledge. Due to the integration of these three aspects, Bayesian statistics can overcome the problems encountered when considering a small number of samples for a single geographical unit and can somewhat overcome the problems encountered when considering the correlations among various geographical units for spatiotemporal data analysis. Given the hierarchical structure of the space-time data of PM_2.5_ concentrations in this paper, a space-time Bayesian hierarchal model (STBHM) [38] that combines a space-time interaction model with BHM was employed. STBHM can estimate the local PM_2.5_ pollution level relative to an overall PM_2.5_ concentration by integrating spatiotemporal PM_2.5_ concentration data. Meanwhile, the local temporal variation trend relative to an overall temporal trend can also be estimated. The goal of employing STBHM is to detect areas with relatively high PM_2.5_ pollution and estimate the local variation trends of PM_2.5_ concentrations.

At the national scale, country-level administrative regions are investigated using a minimum number of analytical units. Pixels with resolutions of 0.1° × 0.1° act as basic analytical units at the urban group level. Collectively, these basic analytical units are referred to as a geographical unit. Here, y_it_ is the observed PM_2.5_ concentration in the geographic unit i = 1, 2, …, for the year t = 1998, 2005, …, 2014. Using the log link function STBHM, this relationship can be expressed as follows:

(1) $$y_{\text{it}}\sim \text{Normal}\left(\mu_{y_{\text{it}}},\sigma_{0}^{2}\right)$$

(2) $$\mu_{y_{\text{it}}}\sim \text{Normal}\left(\theta_{\text{it}},\sigma_{1}^{2}\right)$$

(3) $$\ln\left(\theta_{\text{it}}\right)=\alpha +S_{i}+\left(b_{0}t^{*}+v_{t}\right)+b_{1i}t^{*}+\epsilon_{\text{it}}$$

where $\mu_{y_{\text{it}}}$ and $\sigma_{0}^{2}$ are the average and variance of the PM_2.5_ observations and represent the global variability, $\theta_{\text{it}}$ and $\sigma_{1}^{2}$ are the average and variance of $\mu_{y_{\text{it}}}$, which reflect the differences among the local units. In Formula (3), α is the overall log PM_2.5_ pollution level during the study period in China, and $\text{exp}\left(S_{i}\right)$ is the local magnitude of PM_2.5_ pollution relative to an overall PM_2.5_ pollution. If the posterior estimation of $\text{exp}\left(S_{i}\right)$ is greater than 1.0, the PM_2.5_ pollution level is higher than the national total or vice-versa. $b_{0}t^{*}+v_{t}\;$reflects the overall temporal trend, $t^{*}$ is the centring time during the study period, and $v_{t}$ describes a random temporal effect. $b_{1i}$ represents the local trends of the departure of the geographic unit i from the common temporal variation. A positive estimate of $b_{1i}$ poses a stronger temporal trend than the overall trend and vice-versa. $\epsilon_{\text{it}}$ is the over-dispersion parameter that reflects a certain random effect.

#### 2.2.3. Determinations and Implementation

The coefficient $S_{i}$ of the local magnitude of PM_2.5_ pollution relative to an overall PM_2.5_ pollution and the local trend parameter $b_{1i}$ are assigned by the Besag, York, and Mollie spatial model (BYM) [39]. BYM considers both spatially structured random effects with a convolution algorithm and unstructured random effects using a normal distribution. The spatial structure effects are modelled by using conditional autoregressive (CAR) [40]. The spatial adjacency matrix W adopts the first order “Queen” form. The concrete form is as follows:

(4) $$S_{i}|S_{\left(-i\right)}\sim \text{Normal}\left(\mu_{i}+\overset{n}{\underset{j=1}{\sum }}w_{\text{ij}}\left(S_{j}-\mu_{j}\right),{\;\sigma }_{i}^{2}\right)$$

where $S_{\left(-i\right)}=\left\{S_{\left(j\right)}:j\neq i\right\}$, $E\left(S_{i}\right)=\mu_{i}$, $\text{E}\left(S_{j}\right)=\mu_{j}$, $w_{\text{ij}}$ is the element of spatial adjacency matrix W, and $\sigma_{i}^{2}$ is the variance of $S_{i}$. The location parameters α and $b_{0}$ were assigned to the improper prior distribution, flat(−∞,+∞). Regarding the suggestions of Gelman [41], the standard deviations of the random effects were assigned to the positive half of the Gaussian distribution before N_+∞_ (0.10). The posterior distribution of the model parameters was estimated using Markov chain Monte Carlo (MCMC) simulations. During the Bayesian estimation process, WinBUGS [42] was implemented. The Gelman-Rubin statistic indicator [43] was used to assess the convergence of the model.

## 3. Results

### 3.1. Preliminary Statistics

**Figure 2:**
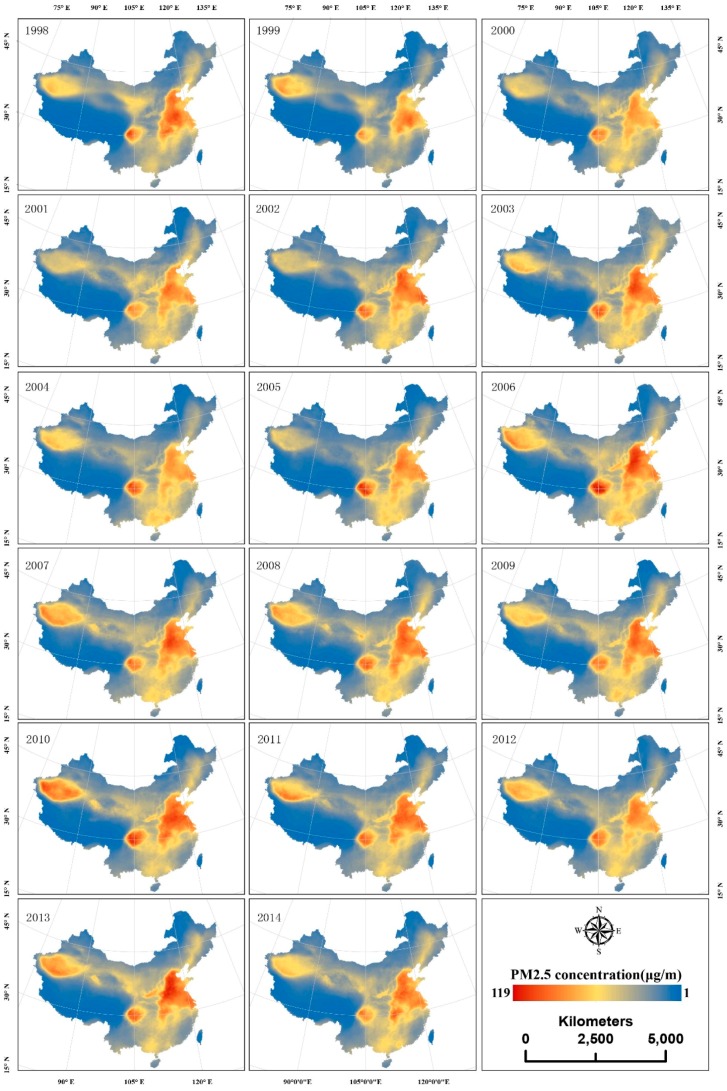
Space-time distribution of the PM_2.5_ concentrations in China from 1998 to 2014.

**Figure 3:**
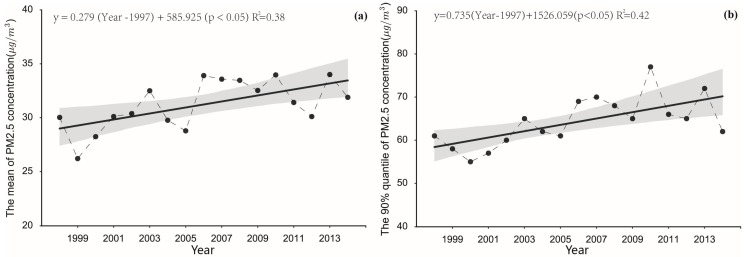
The scatter plots and corresponding linear regression line with 95% CIs of (**a**) the mean and (**b**) the 90% quantiles of the PM_2.5_ concentrations in China from 1998 to 2014.

The spatiotemporal patterns of PM_2.5_ concentrations across China over 17 years (from 1998 to 2014) are illustrated in Figure 2. During the study period, a steady spatial PM_2.5_ concentration distribution formed a “3-Clusters” pattern. The “3-Clusters” represent three areas: the Tarim Basin located in the northwest of Xinjiang Province, the Sichuan Basin located in the southwest, and the North China Plain containing the Jingjiji urban group and Shandong Province. The “3-Clusters” regions had high levels of PM_2.5_ pollution, especially the Sichuan Basin and the North China Plain, which have always had the highest pollution levels in China from 1998 to 2014 (17 years). Regarding the temporal trends in PM_2.5_ pollution, the mean and 90% quantile of the PM_2.5_ concentrations in China generally maintained a statistically significant increasing trend according to the liner regression statistical analysis shown in Figure 3, with increments of 0.279 μg/m^3^ (95% CI: 0.083–0.475) and 0.735 μg/m^3^ (95% CI: 0.261–1.210). The mean minimum and maximum PM_2.5_ concentrations in China occurred in 1999 and 2010 and were 26.2 μg/m^3^ and 33.97 μg/m^3^. Five local peaks in the PM_2.5_ concentrations occurred in 1998, 2003, 2006, 2010, and 2013 during the 17 considered years. In contrast, the PM_2.5_ concentrations were lower in 1999, 2005, and 2012. A large increase was observed to occur from 1999 to 2003, with an increase from 26.2 μg/m^3^ to 32.50 μg/m^3^. The maximum yearly increase from 28.78 μg/m^3^ to 33.91 μg/m^3^ occurred from 2005 to 2006. During 2006–2010, the mean PM_2.5_ concentration over China remained high, with concentrations of between 32.53 μg/m^3^ and 33.97 μg/m^3^.The 90% quantile trend is roughly similar to the mean trend. Integrating the mean and 90% quantile trends showed that the pollution concentrations in China were higher in 2006, 2007, 2010, and 2013. Figure 2 shows that the “3-Clusters” regions all had higher PM_2.5_ concentrations in 2006, 2007, 2010, and 2013.

**Figure 4:**
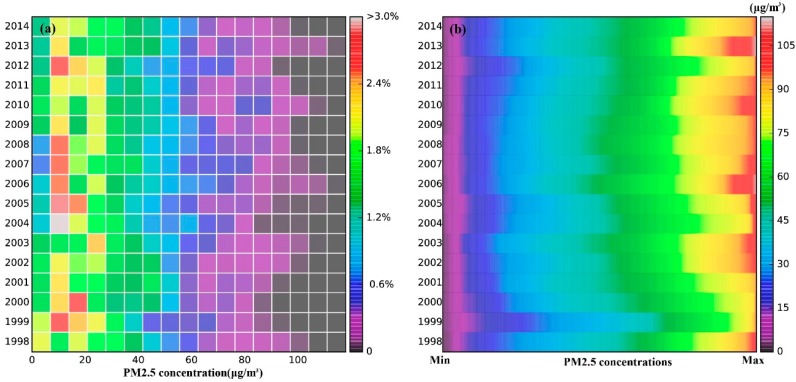
(**a**) Time histogram of the PM_2.5_ concentrations in China by year for 1998–2014; each row illustrates a histogram of the PM_2.5_ concentrations in the corresponding year, and various colours represent the frequency divided by the class width; (**b**) The map of the annual PM_2.5_ concentration ranking grade over China from 1998 to 2014, where each row is the yearly descending rank of the PM_2.5_ concentrations from left to right.

**Figure 5:**
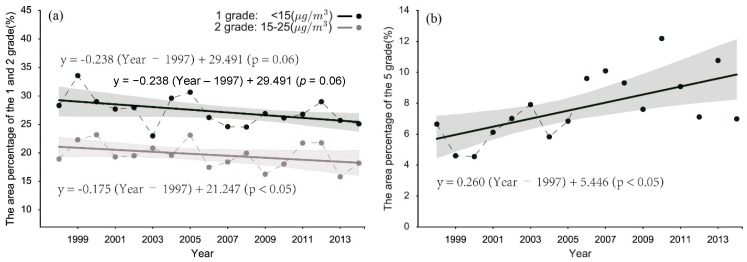
Scatter plots and linear regression lines of the area percentages of (**a**) grade 1 and 2 (<15 μg/m^3^, 15–25 μg/m^3^) and (**b**) grade 5 (>70 μg/m^3^) over China from 1998 to 2014. The upper liner regression equation is the fit of the grade 1 data, and the bottom linear regression equation is the fit of the grade 2 data.

In addition to the mean and 90% quantile, we investigated the variations of the histograms showing the distribution and gradient of PM_2.5_ concentrations over China. Figure 4a shows the temporal variation of PM_2.5_ in a time histogram, where each row represents the corresponding histogram of PM_2.5_ concentrations, and the small squares with different colors reflect different frequencies. Although the annual average PM_2.5_ concentrations over China were mainly distributed from 26.20 to 34.00 μg/m^3^, the area proportion with different PM_2.5_ concentrations grades varied year by year as shown in Figure 4b, meanwhile the linear regression result as shown in Figure 5 demonstrated it. The classification of PM_2.5_ concentrations in this paper primarily refer to the WHO Air quality guidelines for particulate matter [44] and the specific conditions in China. The statistical results explained that the percentages of the areas with PM_2.5_ pollution grades of 1 and 2 were <15 μg/m^3^ and 15–25 μg/m^3^ and roughly decreased, as shown in Figure 5a. In addition, the annual decreasing rates were approximately 0.238 and 0.175 percentage points for the areas classified as pollutant grades 1 and 2 respectively, as shown in Figure 5a. Nevertheless, the highly polluted area, grade 5, with concentrations of >70 μg/m^3^, increased significantly at an annual rate of 0.260 percentage points, as shown in Figure 5b.

**Figure 6:**
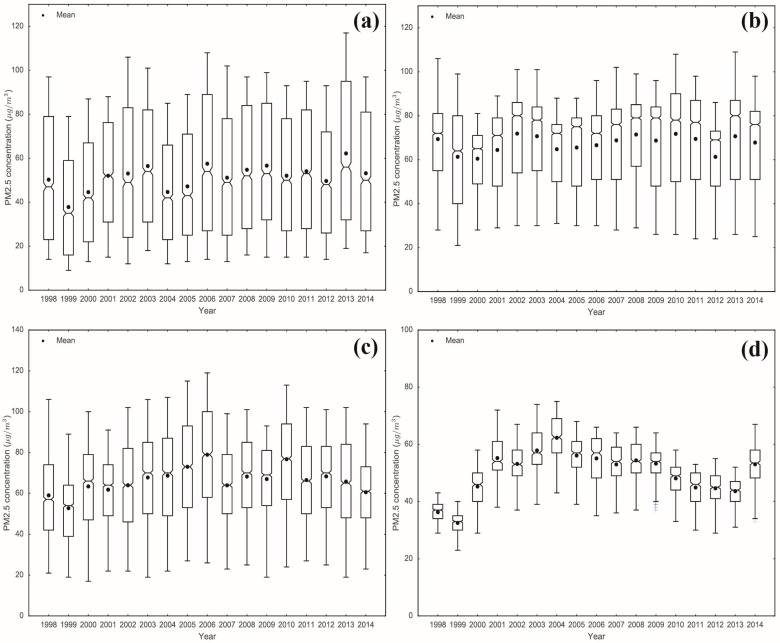
Boxplots of the PM_2.5_ concentrations in the (**a**) Jingjinji urban group; (**b**) Yangtze Delta urban group; (**c**) Cheng-Yu urban group and (**d**) Pearl Delta urban group from 1998 to 2014.

The temporal variations of the PM_2.5_ concentrations in the four urban groups in the Jingjinji, Yangtze Delta, Cheng-Yu, and Pearl Delta in China have their own various features, as shown in Figure 1. Figure 6 illustrates differences in the range and temporal trends among the four city agglomeration regions. 

The 90% quantile, median, and 10% quantile in the Jingjinji urban group increased from 1998 to 2014. The linear regression results show that the annual increment of the median and 10% quantiles of PM_2.5_ concentrations in the Jingjinji urban group are statistically significant {0.586 μg/m^3^ (95% CI: 0.080–1.092) and 0.412 μg/m^3^ (95% CI: 0.098–0.726), respectively}. Meanwhile, the maximum and minimum PM_2.5_ concentrations were observed in the Jingjinji and Pearl Delta urban regions, respectively. The spatial heterogeneity of the PM_2.5_ concentrations was the strongest in Jingjinji. In the Pearl Delta urban group, the spatial difference between the PM_2.5_ concentrations was small. However, the strong increase in the PM_2.5_ concentrations peaked in this region from 1999 to 2004 and then slowly decreased. The remaining two urban group regions, Yangtze Delta and Cheng-Yu, experienced stable and stochastic PM_2.5_ concentration fluctuations.

### 3.2. Bayesian Estimation over China

**Figure 7:**
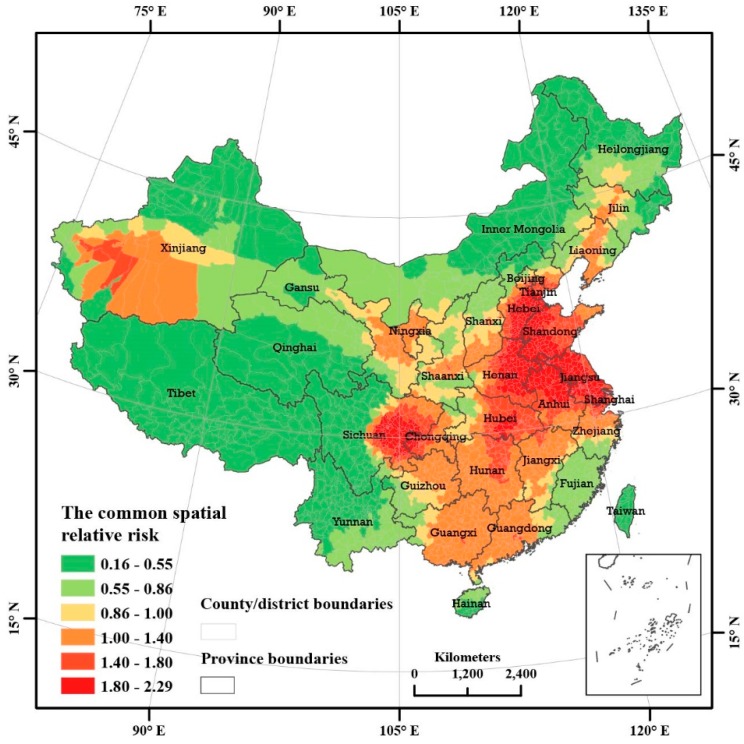
Estimation of the local magnitude of PM_2.5_ pollution relative to the overall PM_2.5_ pollution over China, the posterior medians of $\text{exp}\left(s_{i}\right)$ from STBHM.

The spatial local magnitudes of PM_2.5_ pollution relative to the overall magnitude of PM_2.5_ pollution in China were estimated using the space-time Bayesian hierarchy model employed in this paper. Figure 7 shows the estimated results and the spatial patterns at the country and district level. In this paper, six different categories are classified, as shown in Figure 7, according to the local magnitude of the PM_2.5_ pollution relative to the overall magnitude of the PM_2.5_ pollution based on the geometric interval method. The three areas marked in green and pale yellow represent areas with lower pollution, and the other three areas marked by deep yellow and red represent areas that are considered highly polluted.

The areas with high local PM_2.5_ pollution magnitudes relative to the overall PM_2.5_ pollution magnitude cover 1679 counties and districts, with a population of 949 million, which accounted for 69.3% of the total population based on the 6th National Population Census. Highly polluted areas in China have distinct spatial structures and strong spatial aggregation. Most of the pollution is distributed in one large continuous area and three small areas. The most and second most polluted regions are located in a large and continuous area in two independent spatial clusters. The most polluted areas form an equicrural triangle and circle, which is marked by dark red in Figure 7. Moreover, the highly polluted area revealed a concentric ring gradient geometry with higher concentrations in the centre and lower concentrations toward the edges, as shown in Figure 7.

Furthermore, the local magnitudes of PM_2.5_ pollution in the most seriously polluted areas were more than 1.80 times the overall magnitude of PM_2.5_ pollution in China; thus, in these regions, the PM_2.5_ pollution concentrations were more than 1.8 times the national total concentrations. Among these regions, east China covers a wide area that includes five provinces: Hebei, Shandong, Henan, Jiangsu, and Anhui. In addition, a concentrated region shaped like an equicrural triangle centred within the boundaries of Shandong, Jiangsu, Anhui, and Henan province formed during the 17 investigated years of 1998–2014. This area has very important meaning regarding economic geography because it is located between two developed urban groups: the Jingjinji urban group that includes Beijing and the Yangtze Delta urban group that includes Shanghai. Regarding geometry, two megalopolises, one consisting of Beijing and Shanghai and one consisting of Zhumadian, an industrial city in Henan Province, formed the highly polluted area, which covers an area in the shape of an equicrural triangle with three vertices. The highly polluted areas involved 448 counties and districts with a population of approximately 287 million, which was calculated from the 6th National Population Census and accounted for 21.0% of the total Chinese population.

The Sichuan Basin is the most polluted region in China. The pollution in this region is probably related to the bowl-shaped topography of the basin. The highest spatial range of pollution in the Sichuan Basin is circular, which is consistent with the bowl-shaped geometry of the basin. The region includes 73 counties and districts, with a total population of 46.70 million [34]. The Sichuan Basin covers an area shaped like a concentric ring, with a gradient of high to low pollution extending from the centre to the outer edges of the ring. The area can be divided into three concentric gradient rings, with the innermost circle covering the area with the highest pollution, the second ring covering the area with the second highest pollution, and the third ring covering the area with the lowest pollution. In central Hubei there are 18 counties and districts with a local PM_2.5_ pollution magnitude of more than 1.80 relative to the overall PM_2.5_ pollution magnitude. However, the economy is not developed and the population is not dense in Xinjiang, potentially due to its basin topographic features. Overall, 24 counties and districts exhibit PM_2.5_ pollution levels higher than the national total, and 7 of these counties have local PM_2.5_ pollution magnitudes that commonly exceed 1.40 times the overall PM_2.5_ pollution magnitude.

**Figure 8:**
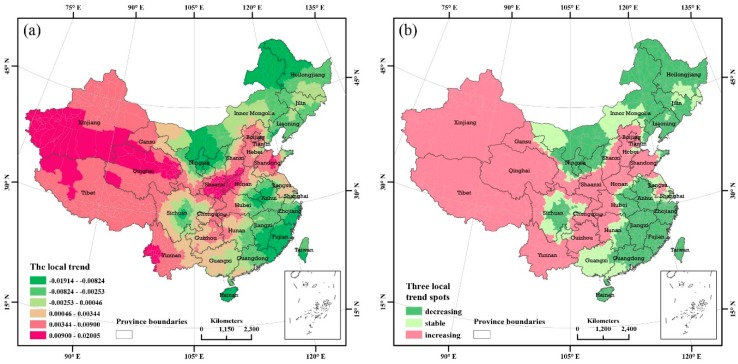
(**a**) Local trend map obtained from the overall trend, the posterior medians of $b_{1i}$ and (**b**) the three local variation clusters classified based on the posterior probabilities of the local trends of more than 0, i.e., $p(b_{1i}>0|\text{data})$ , with stronger variability than the overall trend when $p\left(b_{1i}>0|\text{data}\right)>0.8$ , stable variation when $p\left(b_{1i}>0|\text{data}\right)\leq 0.8$ and $p\left(b_{1i}>0|\text{data}\right)\geq 0.2$ , and weaker variability when $p\left(b_{1i}>0|\text{data}\right)<0.2$ .

In addition to the estimations of the spatial local magnitude of PM_2.5_ pollution relative to the overall magnitude of PM_2.5_ pollution, the STBHM employed in this paper simultaneously estimates the local trends of PM_2.5_ concentrations in each country and district. The local trend is measured by the posterior estimation of the $b_{1i}$ parameter used in the model in this paper. We can distinguish the local trends as follows: (a) a faster increasing trend than the overall trend when the posterior estimated median is $b_{1i}$ > 0 and the posterior probability is $p\left(b_{1i}>0|\text{data}\right)>0.8$; (b) a decreasing trend relative to the common trend when the posterior estimated median is $b_{1i}$ < 0 and the posterior probability is $p\left(b_{1i}>0|\text{data}\right)<0.2$, and (c) a stable trend when the posterior estimated median is $b_{1i}=\;$0 and the posterior probability is $p\left(b_{1i}>0|\text{data}\right)$ and is between 0.2 and 0.8. Figure 8a,b show the estimated local spatial patterns of the PM_2.5_ concentrations in China from 1998 to 2014. Based on the Bayesian estimates of the local trends, we observed that the regions with faster increases than the overall trend covered the large area shown in pink in Figure 8b. In western, eastern, and central China, faster increasing local trends were observed. Specifically, the most polluted area in eastern China, which borders Hebei, Henan, Shandong, and Anhui, shows a faster increasing local trend. Meanwhile, the Xinjiang Tarim Basin area has higher PM_2.5_ pollution and exhibits a faster local trend of increasing pollution. Hence, these two areas should be targeted for preventing and controlling PM_2.5_. In addition, the areas bordering Shannxi, Shanxi, and Henan have experienced strong and local increasing pollution trends.

### 3.3. Bayesian Estimations for the Urban Groups

**Figure 9:**
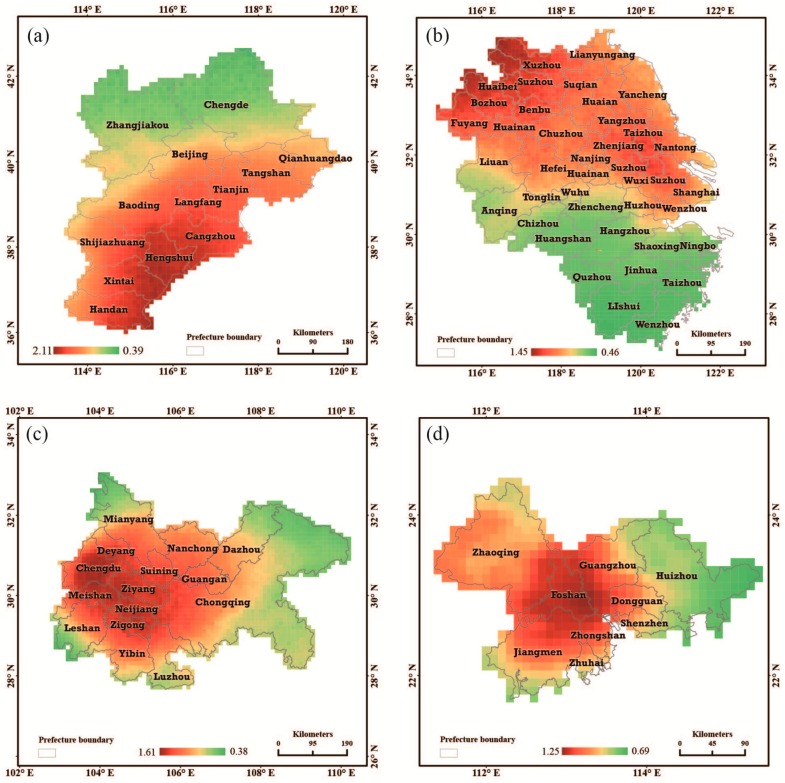
The spatial local magnitude of PM_2.5_ pollution relative to the overall PM_2.5_ pollution in the four urban group areas, and the posterior medians of $\text{exp}\left(s_{i}\right)$ from STBHM for the (**a**) Jingjinji urban group; (**b**) Yangtze Delta Urban group; (**c**) Cheng-Yu urban group; and (**d**) Pearl Delta urban group.

The spatiotemporal variations of the PM_2.5_ concentrations in four urban groups, Jingjinji, Yangtze Delta, Cheng-Yu, and the Pearl Delta urban group, were estimated using the Bayesian hierarchy model in the paper. Figure 9 shows the estimation of the spatial local magnitude of PM_2.5_ pollution relative to the overall magnitude of PM_2.5_ pollution for the areas of the four urban groups in China. For different urban groups, the spatial pattern with relative PM_2.5_ concentrations vary. In the Jingjinji and Yangtze Deltas, the PM_2.5_ concentration distribution distinctly varied from north to south. Nevertheless, in the Cheng-Yu and Pearl deltas, the spatial differentiation radiated outward from the centre. Furthermore, the spatial heterogeneity of PM_2.5_ pollution is highest in the Jingjinji urban group, with minimum and maximum estimated spatial local magnitudes of PM_2.5_ pollution of 0.39 and 2.11 relative to the overall magnitude of PM_2.5_ pollution, respectively. The remaining three urban groups have approximately the same degree of PM_2.5_ pollution heterogeneity.

The regions in the Jingjinji urban group with severe PM_2.5_ pollution are largely located in the southeast. Four cities, Hengshui, Cangzhou, Xintai, and Handan, have the highest PM_2.5_ pollution. Regarding the geographic location, the pollution of PM_2.5_ in Tianjin is more severe than the PM_2.5_ pollution in Beijing when considering the annual average. For Beijing, the spatial pattern of PM_2.5_ pollution is similar to the whole region. In the urban group, Chengde and Zhangjiakou experienced relatively lower PM_2.5_ pollution. In the Yangtze Delta urban group, the northeast region, including Shanghai, has relatively higher PM_2.5_ concentrations. The five cities located north of the border, Xuzhou, Suzhou, Huaibei, Bozhou, and Fuyang, have the relatively highest PM_2.5_ concentrations. The areas south of Anhui province and Zhejiang province had relatively lower PM _2.5_ concentrations. In the Cheng-Yu and Pearl Delta urban group areas, the regions polluted with higher concentrations of PM_2.5_ were located in the centres of the two areas. Four cities in Cheng-Yu, Chengdu, Ziyang, Meishan, and Neijiang had the highest concentrations of PM_2.5_ pollution. In the megacity, Chongqing, which is located in the western region of China and borders highly polluted cities, high concentrations of PM_2.5_ were observed. One of the three areas with the highest PM_2.5_ concentrations in China is located in the Cheng-Yu urban group area. The structural and gradually changing spatial pattern of the PM_2.5_ concentration distribution from the centre of Foshan City and outward to the Pearl Delta urban group was formed.

**Figure 10:**
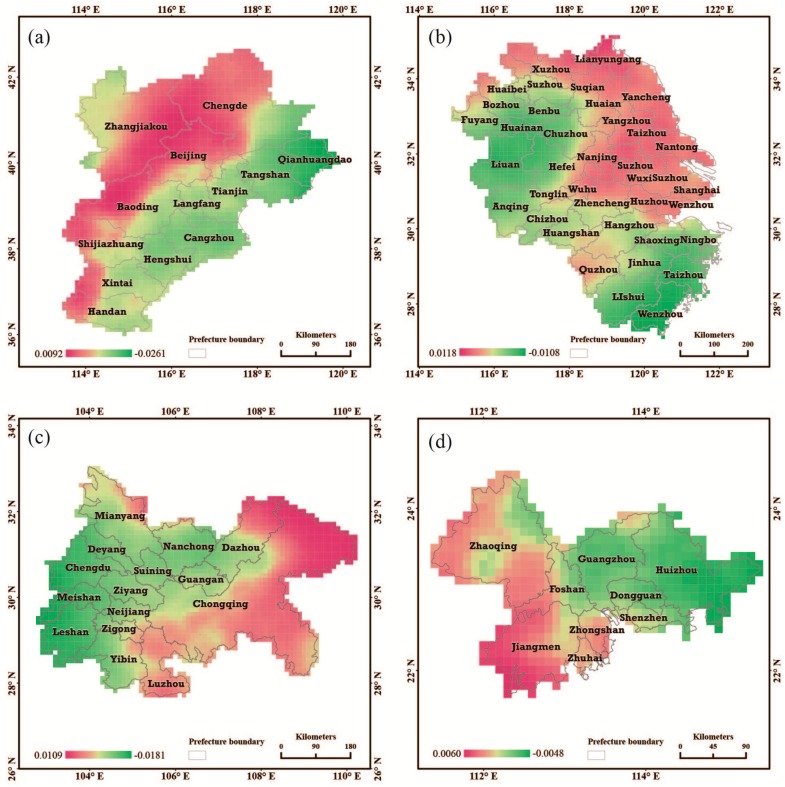
Local trend map relative to the overall trends in the four urban group areas: (**a**) Jingjinji urban group; (**b**) Yangtze Delta Urban group; (**c**) Cheng-Yu urban group; and (**d**) Pearl Delta urban group, and the posterior medians of $b_{1i}$ from STBHM.

To investigate the local temporal variations of PM_2.5_ concentrations in the four urban groups, we estimated their local trends by using the Bayesian model in the paper. The estimated results are shown in Figure 10. As described in the methodology section, if the posterior median of the local trend parameter $b_{1i}$ is greater than 0, the local trend increases faster than the overall trend over the study area. For the estimated results shown in Figure 9, greater increases of $b_{1i}$ above 0 correspond with relatively faster local increases in one urban group area. In the Jingjinji, Cheng-Yu, and Pearl Delta urban group areas, cities with less pollution have inversely higher local increasing trends. Nevertheless, 18 cites, including Shanghai and Nanjing in the urban area of the Yangtze Delta, have experienced not only high PM_2.5_ concentrations but also faster increasing local trends. In other words, these regions will continue to deteriorate if air pollution is not controlled. Thus PM_2.5_ pollution should be prevented and controlled in these 18 cities.

## 4. Discussion

The latest PM_2.5_ concentrations derived from satellite by van Donkelaar et al. [31] were used in this study. Multiple types of satellite remote sensing data, e.g., MODIS Dark Target, MODIS MAIAC, MODIS and SeaWiFS Deep Blue, and MISR, were integrated to produce this dataset. The estimated PM_2.5_ results are highly consistent, with R^2^ = 0.81 for out-of-sample cross-validated PM_2.5_ concentrations from 4082 monitoring points [31]. In North America, East Europe, and Central Europe, low levels of uncertainty with bias of −0.7 to 0.4 μg/m^3^ compared with the monitored PM_2.5_ concentrations were observed. In Latin America and parts of Asia, relatively high uncertainty occurred, with bias of up to 11.6 μg/m^3^ [31]. Considering the high PM_2.5_ concentrations in China, the maximum PM_2.5_ concentration was 117 μg/m^3^, and the precision of the satellite-retrieved PM_2.5_ concentrations was reasonable in scope. Furthermore, Peng et al. [2] confirmed the accuracy of the previous data produced by van Donkelaar for the specific area of China. Peng et al. [2] indicated that the correlation coefficient of remotely sensed PM_2.5_ concentration data against the PM_2.5_ concentration monitored in-situ was 0.79 in China, and the distribution of the residuals’ cumulative probability was random.

China not only has a large population, but also a high population density with unbalanced development. The risk of exposure to PM_2.5_ is particularly prominent in China. Considering that the demographic grid data from 2000 to 2010 was based on the 5th and 6th Chinese Population Census and was more reliable, we selected the population grid data in 2000 and 2010 to discuss exposure to PM_2.5_ in China. According to the statistical results, the increases in the percentage of exposure to PM_2.5_ concentrations greater than 100 μg/m^3^ and 70 μg/m^3^ were the most significant. In 2000, the PM_2.5_ concentration in China did not exceed 100 μg/m^3^; however, the percentage of the population exposed to PM_2.5_ concentrations greater than 100 μg/m^3^ was 5.1% in 2010. The percentage of the population exposed to PM_2.5_ concentrations greater than 70$\;\mu \text{g}/m^{3}$ increased from 23.4% in 2000 to 39.6% in 2010. The exposure ratios of the other PM_2.5_ concentration gradients maintained a relatively stable status. The exposure percentages to PM_2.5_ concentrations greater than three with target WHO concentrations of 35 μg/m^3^, 25 μg/m^3^, and 15 μg/m^3^ were 81.1%, 91.6%, and 98.1% in 2000, and 82.0%, 91.2%, and 98.9% in 2010, respectively.

The results in the paper are some different from those of previous studies. Generally, the PM_2.5_ concentrations across China determined in this paper are lower than those determined in studies conducted by Peng et al. [2] and Ma et al. [28]. However, the proportion of the area with PM_2.5_ concentrations of less than 15 μg/m^3^ is lower than the proportion observed by Peng J.’s, with a difference of −11.3% in 1999 and −7.4% in 2011. The area proportions of the middle PM_2.5_ concentrations of 25–35 are different, with 4.9% in 1999 and −0.6% in 2011. The locations with high PM_2.5_ pollution detected by STBHM in this paper are roughly consistent with those identified in previous studies. Based on the overall and local trends, we have drawn various conclusions. The estimated results in this paper indicated that the PM_2.5_ concentrations over China generally increased from 1998 to 2014, while Ma et al. [28] identified two stages, an increasing stage from 2004 to 2007 and a decreasing stage from 2008 to 2013. The estimates in our study suggested that the entire western area experienced a local increasing trend. Peng et al. [2] noted that only the Xinjiang area showed a gradually increasing local trend and that the remaining regions in the western area maintained a stable local trend.

Considering the topographic features, “3-Clusters” belonged to a local low-lying area, indicating that the annual average PM_2.5_ concentration is related to regional terrain characteristics. In Low-lying areas, PM_2.5_ generally aggregates easily, while the impact of regional wind appears weaker in terms of the entire year. The Sichuan Basin and the North China Plain regions have higher population densities. We suspect that the PM_2.5_ concentration is also related to population density. Han et al. [45] illustrated a significant correlation between PM_2.5_ concentration and population in Beijing.

The PM_2.5_ concentrations in China showed a statistically significant increasing trend, with increments of 0.279 μg/m^3^ (95% CI: 0.083–0.475) and 0.735 μg/m^3^ (95% CI: 0.261–1.210). The areas with low PM_2.5_ pollution are shrinking, while the areas with high PM_2.5_ pollution are expanding. These changes suggest that the environmental governance in China should be continuously strengthened. The areas of focus are the North China Plain and the Sichuan Basin regions, which have high PM_2.5_ concentrations and the same populations. Based on Bayesian estimations, the areas with the most severe pollution, with a common spatial relative risks of more than 1.0, covered 1679 counties or districts and affected a population of 949 million people, accounting for 69.3% of the total population. The western, eastern, and central regions of China all exhibited faster increasing local trends than the overall trend. It should be noted that the North China Plain regions have high PM_2.5_ pollution levels and exhibit increasing local trends. In other words, the PM_2.5_ pollution in the North China Plain regions can be expected to further deteriorate if no effective preventative measures are implemented. Although the western regions in China have lower PM_2.5_ pollution levels, increasing trends have been occurring.

This paper primarily focused on the spatiotemporal variations of PM_2.5_ pollution in China in four large urban areas and has the following limitations. First, on-site monitored PM_2.5_ concentration data were not used in this study. The research results from our study are based on remotely sensed PM_2.5_ concentrations. If remote sensing inversion and on-site measured data are integrated together, the results will be more reliable. Second, because the STBHM requires heavier computation, the county-level or district-level administrative regions were considered as a statistical unit when detecting the spatiotemporal variability of the PM_2.5_ concentrations at the national scale. Thus, the average pooling process had to be executed before calculation, which could add some uncertainty. Third, this paper mainly focused on the space-time variability of PM_2.5_ concentrations and did not provide the influencing mechanism of PM_2.5_ pollution. However, we hope that this paper provides new insights for considering PM_2.5_ pollution. In the future, we will collect data from more PM_2.5_ data sources, e.g., on-site monitoring data, to continue studying the dynamic process of PM_2.5_ pollution based on multi-source data. In addition, promoting the efficiency of the STBHM for applications with remote sensing pixel data is an important area for future research, and the influencing factors and mechanisms of PM_2.5_ pollution should be an important area of concern.

## 5. Conclusions

The spatiotemporal patterns and trends of PM_2.5_ concentrations in China during 1998–2014 are analysed in this paper. The related remotely sensed dataset is the latest version of the inverted data produced by the team lead van Donkelaar. First, STBHM is employed to analyse the spatiotemporal variations of the PM_2.5_ concentrations in China from 1998 to 2014. Meanwhile we briefly discussed the status of the exposure to PM_2.5_ concentrations at the national level. Our study primarily arrived at the conclusions presented below:

Generally, a steady “3-Clusters” spatial pattern of high PM_2.5_ pollution formed. The “3-Clusters” areas, the Tarim Basin region located in the northwest, the Sichuan Basin region located in the southwest, and the North China Plain containing the Jingjiji urban group and Shandong Province, experienced higher PM_2.5_ pollution, particularly in the latter two cluster regions.

Different large urban areas showed different spatial PM_2.5_ pollution structures, with the northern and southern areas showing differences in the Jingjinji and Yangtze Deltas and a circular PM_2.5_ concentration gradient in the Cheng-Yu and Pearl Deltas. The spatial heterogeneity of the Jingjinji urban group was the strongest. The 18 cities, including Shanghai and Nanjing in the Yangtze Delta urban group area, experienced high PM_2.5_ concentrations and the fastest increasing local trends simultaneously. These 18 cities have recently become more developed over China but have resulted in environmental pollution problems. The developed industrialized cities that experience high PM_2.5_ pollution should implement industry transformations.
